# Diagnosis and surgical management strategy for pediatric small bowel obstruction: Experience from a single medical center

**DOI:** 10.3389/fsurg.2023.1043470

**Published:** 2023-02-21

**Authors:** Mingzhu Liu, Fengchun Cheng, Xijie Liu, Bufeng Zheng, Feifei Wang, Chengwei Qin, Guojian Ding, Tingliang Fu, Lei Geng

**Affiliations:** Binzhou Medical University Hospital, Binzhou, China

**Keywords:** small bowel obstruction, intestinal ischemia, diagnosis, management, pediatric

## Abstract

Identifying Bowel strangulation and the approach and timing of surgical intervention for pediatric SBO are still uncertain. In this study, 75 consecutive pediatric patients with surgically confirmed SBO were retrospectively reviewed. The patients were divided into group 1 (*n* = 48) and group 2 (*n* = 27) according to the presence of reversible or irreversible bowel ischemia, which was analyzed based on the degree of ischemia at the time of operation. The results demonstrated that the proportion of patients with no prior abdominopelvic surgery was higher, the serum albumin level was lower, and the proportion of patients in which ascites were detected by ultrasonography was higher in group 2 than that in group 1. The serum albumin level was negatively correlated with ultrasonographic findings of the fluid sonolucent area in group 2. There were significant differences in the choice of surgical approach between group 1 and group 2. A symptom duration of >48 h was associated with an increased bowel resection rate. The mean length of hospital stay was shorter in group 1 than that in group 2. In conclusion, immediate surgical intervention should be considered in patients with a symptom duration of >48 h or the presence of free ascites between dilated small bowel loops on ultrasonography. Laparoscopic exploration is recommended as first-line treatment in patients with stable status.

## Introduction

Pediatric small bowel obstruction (SBO) is a common surgical emergency, presenting symptoms and signs similar to those seen in other causes of acute abdomen (1, 2). If there is a delay in the diagnosis and proper management of SBO, intestinal strangulation or ischemia may occur, resulting in high morbidity and mortality rates (1–4). However, approximately one-third of strangulated SBO cases present with reversible ischemia (5). Although there are no reliable parameters for predicting reversible or irreversible intestinal ischemia, it is necessary to minimize the chance of overlooking strangulation in managing SBO (3, 6, 7). Therefore, accurate identification and timely treatment of strangulated SBO are vital for preserving the ischemic intestine and avoiding life-threatening conditions (8). Herein, we retrospectively reviewed pediatric patients with acute SBO admitted to our medical center between January 2007 and December 2021. Clinical data were analyzed, focusing on the predictors of strangulated SBO, the timing of surgical intervention, and the laparoscopic management strategy.

## Methods

Clinical data of 75 children with SBO admitted to Binzhou Medical University Hospital between January 2007 and December 2021 were analyzed. The inclusion criteria are as follows: (1) abdominal pain, vomiting, complete cessation of defecation; (2) abdominal tympany, tenderness, and evidence of focal or generalized peritonitis; (3) abdominal radiography results revealing air-fluid levels, dilated bowel loops, and paucity of rectal gas (9); (4) point-of-care ultrasound (POCUS) demonstrating a dilated intestinal loop (>2.5 cm) and abnormal intestinal peristalsis (5); (5) history of urgent and emergency surgical procedures; and (6) age 0–18 years. The exclusion criteria are as follows: (1) age >18 years; (2) history of conservative management; (3) congenital intestinal anomalies, including malrotation; and (4) gastrointestinal tumors.

Variables including demographics, symptoms and signs, laboratory findings [white blood cell (WBC) count, albumin level, and sodium level], imaging findings (abdominal radiography, POCUS, or CT scan), and length of hospital stay were collected retrospectively. Symptom duration was defined as the period from symptom onset to anesthesia completion. Abdominal pain was defined as tenderness and/or subjective evidence of pain.

The surgical indications included fever, tachycardia, leukocytosis, and localized or generalized abdominal tenderness (10); (2) hypovolemic or septic shock; (3) abdominal imaging findings suggestive of abdominopelvic effusion or mesenteric edema (4, 11); (4) a symptom duration of ≥48 h; and (5) failed conservative management.

The patients were divided into the reversible intestinal ischemia and irreversible intestinal ischemia groups (groups 1 and 2, respectively) according to intraoperative findings for the analysis. Simple adhesive lysis was performed in group 1, whereas segmental intestinal resection was required in group 2. Irreversible intestinal ischemia was defined as bowel necrosis, which was assessed based on the color of the intestinal wall, pulsation of the mesenteric arteries, and peristalsis (11–13).

Differences between the two groups were analyzed using IBM SPSS statistical software (version 26.0; IBM Corp., Armonk, NY, USA). The chi-square test and Student's *t*-test were used to perform the statistical analysis. Statistical significance was set at *P* < 0.05.

## Results

A total of 75 pediatric patients with SBO were enrolled in this study. The median age was significantly higher in group 1 [4.0 years (IQR: 0.94–9.25 years)] than that in group 2 [0.83 years (IQR: 0.42–4.0 years); *P* = 0.014]. The male-to-female ratio was 2:1. Patients with a history of abdominopelvic surgery in group 1 were less likely to undergo small bowel resection than those in group 2 (*P* = 0.005). There were no significant differences in sex (*P* = 0.314), WBC count (*P* = 0.713), NLR (*P* = 0.089), or serum sodium level (*P* = 0.084) between the two groups. The serum albumin level was significantly lower in group 2 than that in group 1 (44.08 ± 6.23 vs. 40.88 ± 5.34, *P* = 0.029), as shown in Table 1.

**Table 1:** Detailed data in the present case series.

	Group 1 (*n* = 48)	Group 2 (*n* = 27)	All (*n* = 75)	*P*-value
Age (year) (%)				0.093
<3	18 (37.50)	18 (66.67)	36 (48.00)	
3–6	12 (25.00)	5 (18.52)	17 (22.67)	
6–9	5 (10.42)	1 (3.7)	6 (8.00)	
>9	13 (27.08)	3 (11.11)	16 (21.33)	
Median age (IQR)	4.0 (0.94–9.25)	0.83 (0.42–4.0)	3.0 (0.54–7.0)	0.014
Sex (%)				0.314
Male	34 (69.56)	16 (59.26)	50 (66.67)	
Female	14 (30.44)	11 (40.74)	25 (34.25)	
Male:female ratio	2.43: 1	1.45: 1	2:1	
Prior abdominopelvic surgery (%)	** **	** **	** **	0.005
Yes	25 (52.08)	5 (18.52)	30 (40.00)	
No	23 (47.92)	22 (81.48)	45 (60.00)	
Symptom duration (%)	** **	** **	** **	0.001
<48 h	32 (65.22)	7 (25.93)	39 (52.00)	
≥48 h	16 (34.78)	20 (74.07)^a^	36 (48.00)	
Laboratory findings
WBC (×10^9^/L)	12.85 ± 6.01	12.26 ± 7.83	12.64 ± 6.07	0.713
NLR	6.48 ± 7.08	3.93 ± 3.78	5.54 ± 6.12	0.089
Albumin (g/L)	44.08 ± 6.23	40.88 ± 5.34	42.88 ± 6.07	0.029
Sodium (mmol/L)	135.06 ± 4.77	133.02 ± 4.65	134.32 ± 4.80	0.084
Imaging examination (%)
POCUS	39 (81.25)	25 (92.59)	64 (85.33)	0.187
Dilated bowel	31 (79.49)	23 (92.00)	54 (84.38)	0.184
Fluid sonolucent area	15 (38.46)	19 (76.00)	34 (53.13)	0.003
CT scan	8 (16.67)	2 (7.41)	10 (13.33)	0.267
Surgical approach (PAS)				0.04
Laparoscopy	28 (17)	0 (0)	28	
Laparoscopic assisted	14 (6)	16 (5)	30	
Conversion	4 (1)	6 (0)	10	
Laparotomy	2 (1)	5 (0)	7	
Length of hospital stay (d)	10.86 ± 3.28	12.77 ± 4.25	11.58 ± 3.76	0.035

yr, year; h, hour; d, day; IQR, interquartile range; WBC, white blood cell; NLR, neutrophil-to-lymphocyte ratio.

^a^ There is a significantly higher bowel resection rate than patients whose symptom duration <48 h, *P* < 0.001.

In group 2, fluid sonolucent areas between dilated small bowel loops were detected by POCUS in 19 of 25 patients (76%), while in group 1, they were detected in only 15 of 39 patients (38.46%), *P* = 0.003. Fifty-four cases suggestive of intestinal ischemia were confirmed during surgical exploration in 64 patients who underwent POCUS. The serum albumin level was negatively correlated with ultrasonographic findings of the fluid sonolucent area for the total patient population or group 2 (*r* = −0.5587, *P* = 0.0006; and *r* = −0.6630, *P* = 0.002, respectively). However, there was no correlation between the serum albumin level and bowel dilatation for the total patient population, for group 1, or for group 2 (*P* = 0.5794, *P* = 0.9362, and *P* = 0.9085, respectively).

The overall bowel resection rate due to irreversible intestinal ischemia was 36% (27/75), with a higher rate of bowel resection in patients with a symptom duration of greater than 48 h (17.95% vs. 55.55%, *P* < 0.001). The etiology, prior abdominopelvic surgery, and surgical approaches are listed in Table 2. Congenital or acquired adhesions were the most common causes of SBO (44/75, 58.67%). The second-most common cause was intussusception (15/75, 20%).

**Table 2:** Etiology of pediatric SBO patients with or without prior abdominopelvic surgery.

Etiology and surgical approach	Group 1 (*n* = 48)	Group 2 (*n* = 27)
Cause in patients with PAS	25	5
Peritoneal adhesion	19	5
Internal hernia	4	0
Fecal stone	2	0
**Prior abdominopelvic surgery**
Appendectomy	8	2
Open reduction of intussusception	8	1
Soave procedure	1	2
Ladd procedure	4	0
Transverse colectomy	2	0
Intestinal anastomosis due to meconium peritonitis	1	0
Splenic autotransplantation after splenectomy	1	0
Kasai procedure	1	0
Cause in patients without PAS	23	22
Congenital fibrotic bands	5	4
Intussusception	7	8
Appendicitis	2	2
Volvulus without malrotation	3	4
Meckel diverticulum	3	4
Internal hernia	1	1
Fecal stone	1	0
Peritoneal tuberculosis	1	0

SBO, small bowel obstruction; PAS, prior abdominopelvic surgery.

**Figure 1:**
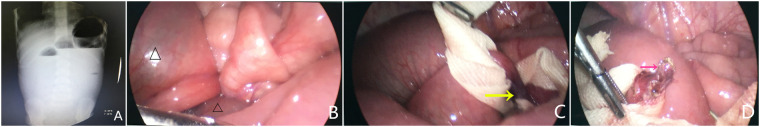
Plain abdominal radiography and intraoperative findings revealed complete SBO (**A**), dilated small intestine (**B**, △), a single adhesive band (**C**, yellow arrow), and adhesiolysis completion (**D**, pink arrow) in a 6-year-old boy who had undergone a prior appendectomy due to acute perforated appendicitis.

**Figure 2:**
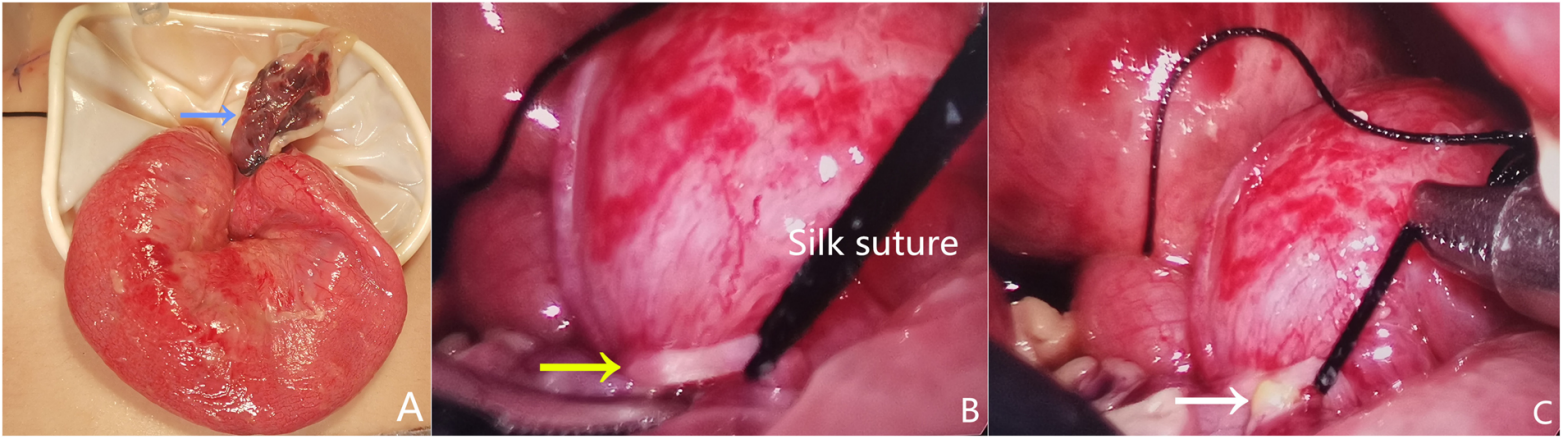
Intraoperative findings revealed a necrotic Meckel diverticulum and laparoscopic transient occlusion (**A**, blue arrow) associated with a concomitant fibrotic band (**B**, yellow arrow) and occult perforation (**C**, white arrow) in a 5-month-old boy with sepsis.

There were significant differences in the choice of surgical approach between groups 1 and 2 (*P* = 0.04). In group 1, 46 of 48 patients underwent laparoscopic exploration, including 28 laparoscopic procedures (Figure 1), 14 laparoscopic-assisted procedures (Figure 2), and 4 conversion procedures. In group 2, 22 of 27 patients underwent laparoscopic surgery, including 16 laparoscopic-assisted procedures and 6 conversion procedures.

Postoperative minor dehiscence of mini-incision occurred in one infant with diffuse peritonitis and severe sepsis. One patient required reoperation due to recurrent adhesive SBO after undergoing laparoscopic-assisted mini-incision adhesiolysis. The mean length of hospital stay was significantly lower in group 1 than that in group 2 (10.86 ± 3.28 d vs. 12.77 ± 4.25 d; *P* = 0.035).

Postoperative minor dehiscence of mini-incision occurred in one infant with diffuse peritonitis and severe sepsis. One patient needed reoperation due to reoccurring adhesive SBO after laparoscopic-assisted mini-incision adhesiolysis. The mean length of hospital stay was significantly smaller in group 1 than that in group 2 (10.86 ± 3.28 d vs. 12.77 ± 4.25; *P* = 0.035).

## Discussion

SBO is one of the most common acute abdominal surgeries performed in pediatric patients (14). In this study, the male-to-female ratio was 2:1, which is similar to that in other reports in the literature (15, 16). Congenital or acquired adhesions were the most common cause of SBO, with an incidence of 58.67%, which is consistent with previous reports (17–19). Other causes of SBO included irreducible intussusception, intestinal volvulus without malrotation, and fecalith obstruction. However, in the adult population, the most common causes of SBO are herniation, malignancy, and postoperative or inflammatory adhesive disease. Intussusception, Crohn's disease, gallstone ileus, intestinal volvulus, internal hernia, foreign bodies, and bezoars must also be considered in the differential diagnosis. Moreover, approximately 65%–75% of SBO cases in developed countries are due to postoperative intra-abdominal adhesions (14, 20–25). In our case series, only 40% of the patients had undergone prior abdominopelvic surgery.

Our results also demonstrated that the prevalence of irreversible ischemia was 36%, which is consistent with that reported in previous studies (16%–33%) (15, 26, 27). However, younger children are more likely to develop irreversible intestinal ischemia. Moreover, 80% of patients experienced irreversible bowel ischemia without prior abdominopelvic surgery, suggesting a delayed diagnosis and management in younger children with a virgin abdomen (13, 14, 23).

Regarding an early and accurate diagnosis, a detailed medical history, repeated clinical examination, laboratory parameters, and imaging are essential to identify intestinal ischemia in pediatric SBO (28). It is crucial for surgeons to differentiate between patients who can be safely treated nonoperatively and those who need urgent and emergency surgical intervention (29) due to a delay in surgery, which may lead to intestinal strangulation, ischemia, and even necrosis. However, nontherapeutic laparotomy may also result in an additional risk of adhesion formation and exposure to anesthesia (30). Moreover, clinical examination for identifying bowel strangulation or ischemia has a relatively low sensitivity (24). The value of systemic inflammatory factors, including C-reactive protein (31) and procalcitonin (32), in evaluating strangulated SBO remains uncertain. The value of plain radiography is also unable to reliably detect the early signs of intestinal strangulation (31). Bouassida et al. (33) created a score to detect potential bowel ischemia, exhibiting a high accuracy rate. This predictive score, which takes into consideration the patient's age, symptom duration, body temperature, WBC count, reduced bowel wall enhancement, and/or mesenteric fluid on a CT scan, may help surgeons decide on emergency surgery in those with bowel ischemia. Our data revealed that there was a significantly lower serum albumin level in the irreversible ischemia group, and the presence of free fluid detected by POCUS was negatively correlated with a lower serum albumin level in the total patient population and in group 2, which may reflect the acute severe condition of these patients.

POCUS can detect a dilated fluid-filled intestinal loop more than 2.5 cm in diameter and abnormal intestinal peristalsis in patients with SBO (5, 34). Considering the advantages of POCUS, such as its convenience and repeatable assessment of SBO with high sensitivity and specificity without radiation exposure, it is widely used in pediatric patients with the suspected surgical acute abdomen (17, 32, 35). In the present study, free fluid between the dilated small bowel loops detected by POCUS may be suggestive of worsening SBO (36). Among the 64 patients who underwent POCUS, 54 underwent emergency surgery and confirmed intestinal ischemia, suggesting that it exhibits good accuracy in identifying bowel ischemia.

CT is a useful tool for diagnosing SBO in adults. It can help identify the signs of bowel ischemia, including bowel wall enhancement, mesenteric edema or engorgement, and fluid or free air in the peritoneal cavity (37, 38). This “transition zone” may indicate the site of SBO (4, 29). It is challenging to identify patients with nonstrangulated bowel obstruction for whom initial nonoperative management may fail to avoid delayed surgical intervention (37). However, Tresallet et al. (39) recommended that CT should not be routinely considered a diagnostic tool in clinical decision-making because it is less accurate in predicting emergency surgery.

Regarding the management of pediatric SBO, the findings of a physical examination and physiologic parameters are still the gold standard for clinical decision-making in emergency surgical intervention or initial nonoperative management (13). If SBO progresses to complications such as bowel ischemia, perforation, severe physiological disorders, and sepsis, especially in the pediatric population, any delay in surgical intervention may result in a high risk of morbidity and mortality (33, 37). Once a patient presents with suspected SBO, an early surgical evaluation should be performed (6, 20). Conservative treatment, including fasting, nasogastric suction, and fluid therapy, is the first choice in adult patients, and it exhibits a success rate of 16%–63% (17, 26, 40). However, approximately 80% of children with SBO require surgical intervention because of the high risk of bowel strangulation, especially in those undergoing initial conservative management for >48 h (10, 13, 41, 42).

In recent years, Linden et al. (43) reported implementing an enteral water-soluble contrast challenge for pediatric acute SBO after hospitalization, suggesting its dual role in diagnosis and nonoperative management with significant cost savings, decreased surgical intervention, and decreased length of hospital stay. However, Feigin et al. (27) and Hyak et al. (15) suggested that 48 h of symptom duration should be considered the key point for surgical decision-making. Batebo et al. (17) and Lautz et al. (18) demonstrated that children with SBO undergoing surgical intervention within 24 h of symptom onset have fewer occurrences of irreversible intestinal ischemia and postoperative complications. Children with SBO presenting with lethargy, persistent abdominal pain, abdominal distension, and peritoneal irritation signs, which are strongly suggestive of bowel strangulation, usually require emergency surgery (44). Our data revealed a significantly higher bowel resection rate in patients with a symptom duration of ≥48 h. Early operative intervention for pediatric patients with SBO may avoid unnecessary bowel resection and reduce perioperative morbidity and mortality (28).

Regarding the surgical approach for SBO, the Working Group on ASBO of the World Society of Emergency Surgery recommends that laparoscopic surgery should be attempted in patients with a first episode or shorter duration of symptom onset, simple adhesion, stable hemodynamics, no abdominal distension, or peritonitis (11, 25, 31, 45–47). A faster recovery (49) and reduced adhesive formation are the main advantages of laparoscopic exploration (20, 24, 48, 50). However, occult small bowel perforation and signs of ischemia should be carefully examined, as shown in Figure 2. Additionally, the risk of bowel injury can diminish with increased experience. Energy-based devices to divide adhesive bands should be carefully used to avoid bowel injury, as shown in Figure 1. Conversion may be helpful in representing good clinical judgment in some cases (51). In our case series, a necrotic Meckel's diverticulum associated with a concomitant fibrotic band that caused an occult perforation in a child was confirmed by laparoscopic exploration. After laparoscopic occlusion of the base of the diverticulum, adhesiolysis, and thorough peritoneal irrigation, mini-incision was performed for primary intestinal anastomosis.

For patients with multiple previous abdominopelvic procedures, a longer symptom duration, suspected bowel necrosis, or diffuse peritonitis, laparoscopic assistance and/or conversion are required to manage the complicated condition (51). In the present study, more than half of the patients who initially underwent laparoscopic surgery required laparoscopic-assisted mini-incision. Conversion was required in 10 cases owing to marked bowel dilatation, extensive adhesions, or poor visualization, as described in the literature (25, 46, 48). Five of seven patients who underwent initial laparotomy as the first choice were confirmed to have hemodynamic instability, severe shock, irreversible intestinal ischemia, or bowel volvulus.

### Limitations

This retrospective study, which has a relatively small sample size, may be prone to an incomplete collection of individual data. The treatment options may have been affected by the training of pediatric surgeons or their previous experiences with similar cases. All patients were treated at a single medical center, so the results may not be representative of those of other medical centers. Further prospective studies are needed to establish specific recommendations.

## Conclusion

Identifying patients with SBO who require urgent operative intervention or who fail under conservative management is an ongoing challenge (52–54), especially in the pediatric population. Immediate surgical intervention should be considered in patients with symptoms of SBO lasting for >48 h or in those exhibiting free ascites between the dilated small bowel loops on ultrasonography. Laparoscopic exploration is recommended as first-line treatment in patients with stable status. A laparoscopic-assisted mini-incision laparotomy is an option for the resection of necrotic bowel segments or to prevent iatrogenic intestinal injury. The development of predictive models for strangulated SBO and evaluation of the risk/benefit ratios of different management strategies, especially in younger children, are needed (13, 55).

## Data Availability

The raw data supporting the conclusions of this article will be made available by the authors without undue reservation.
